# Evaluating opportunities for improved orthopedics outpatient satisfaction: an analysis of Press Ganey® Outpatient Medical Practice Survey responses

**DOI:** 10.1186/s13018-020-1567-1

**Published:** 2020-01-28

**Authors:** Andrew R. Stephens, Tyson J. Rowberry, Andrew R. Tyser, Nikolas H. Kazmers

**Affiliations:** 1 University of Utah, School of Medicine, 30 N 1900 E, Salt Lake City, UT 84132 USA; 2 Department of Orthopaedics, University of Utah, 590 Wakara Way, Salt Lake City, UT 84108 USA

**Keywords:** Patient satisfaction, Press Ganey, Reimbursements

## Abstract

**Introduction:**

The Press Ganey® Outpatient Medical Practice Survey (PGOMPS) is composed of 10 provider-specific and 15 non-provider-specific questions. Some healthcare systems link PGOMS overall scores to physician reimbursements. The aim of this study was to determine the frequency of patient satisfaction across individual PGOMPS question, the null hypothesis being that there was no variability between the frequency of satisfaction and similar questions.

**Methods:**

We reviewed all new patient orthopedic PGOMPS scores between January 2014 and December 2017. Due to the large ceiling effect, satisfaction was defined as a perfect total score. The frequency of perfect scores for each question was calculated.

**Results:**

Five thousand one hundred sixty-three patients met the inclusion criteria. Two thousand two hundred sixty-six (43.89%) provider-specific questions received perfect satisfaction versus 986 (19.10%) with perfect satisfaction for non-provider-specific questions (*p* <  0.001). The five questions most likely to receive perfect satisfaction were MD friendliness/courtesy (80.36), MD spoke using clear language (80.35%), likelihood to recommend practice (79.11%), likelihood to recommend MD (78.8%), and MD confidence (78.74%). The five least likely were convenience of office hours (60.44%), ease of getting on phone (59.72%), ability to get desired appointment (59.50%), wait time (54.63%), and information about delays (53.80%).

**Conclusions:**

Our results suggest that the majority of orthopedic patients are satisfied with their provider, demonstrating that room for improvement is limited with provider-specific areas. Leaders of health care teams should consider these results when seeking to improve patient satisfaction scores and determining how and if scores should be linked to reimbursements.

## Introduction

The measurement of patient satisfaction has become an integral component of health care over the past two decades. The Patient Protection and Affordable Care Act enabled Medicare to make incentive reimbursements based on the patients’ experience of care [1]. The ability of patient satisfaction scores to directly impact physician compensation has received much evaluation and critique over the past decade [2–5]. A plethora of evidence in the literature has described factors that affect patient satisfaction that are unlikely to be representative of the value of care delivered. Examples of these include patient age [6–8], education level [9, 10], race [9, 11–14] and sex [8, 13–15], provider race [12, 16–18] and sex [19, 20], distance traveled to appointment [6, 21], setting/location of the appointment [9, 13, 22, 23], patient psychological status [24], and time between appointment and completion of survey [25].

The Press Ganey® Outpatient Medical Practice Survey (PGOMPS) is a metric of patient satisfaction and is widely used throughout many health care systems. The survey is composed of 2 wait time questions and an additional 25 questions about the clinic experience: 10 specifically relate to the provider, while the remainder evaluate other components such as, access to care, staff, and the office in general [26]. Most previously published studies have analyzed patient satisfaction at the overall PGOMPS score level. Little has been done to evaluate individual PGOMPS questions. To our knowledge, no prior work has conducted a frequency analysis of all 25 PGOMPS questions in orthopedics. Doing so would provide greater insight into the needs of orthopedic patients and provide providers and health care administrators with specific areas they can focus on to improve a patient’s overall experience. The purpose of our study was to evaluate the patient satisfaction with each individual PGOMPS question and to determine if there is a difference between provider-specific questions and the other questions combined. Our null hypothesis is that there would be no difference in the frequency of satisfaction for each individual question.

## Methods

In an effort to improve the value of care a patient receives and enhance their overall experience, our institution has contracted with Press Ganey Corporation to measure patient satisfaction scores in the outpatient setting. The survey link is sent to patients via email after their visit and a repeat email is sent if the patient has not completed the survey after 5 days. Patients are able to complete the survey within 30 days of receiving the initial email. Previous work published by our institution has shown the response rates of PGOMPS administered electronically to range from 8.9 to 16.5% [26, 27]. Each individual PGOMPS question is measured on a 5-point Likert scale, with 1 indicating very poor and 5 indication very good. Responses are converted to a 100-point scale.

PGOMPS evaluates multiple different aspects of care: access of care (5 questions), moving through your visit (2 questions), nursing (2 questions), provider (10 questions), personal issues (4 questions), and overall care (2 questions). The provider-specific questions include MD friendliness/courtesy, MD explained problem or condition, MD concern for patient questions/worries, MD effort to include patient in decisions, MD information about meds, MD follow-up care instruction, MD spoke using clear language, MD time spent with patient, confidence in MD, and likelihood to recommend MD. Non-provider-specific questions were defined as all questions except for the provider-specific and wait time questions.

Our IRB-approved study retrospectively reviewed patients who prospectively completed the PGOMPS between January 2014 and December 2017 at our orthopedic clinic. Exclusion criteria included any postoperative or follow-up visits. For patients with multiple new visits during the study period with several providers, only the first visit was included. Perfect satisfaction was defined as receiving a 100 on a question given the high ceiling effect of the survey [6, 26, 27]. A frequency analysis of patients giving perfect satisfaction for each individual question (e.g., a perfect total score) was calculated. A frequency analysis was also performed between sexes and age groups defined a priori (< 18, 19–29, 30–39, 40–49, 50–59, 60–69, and 70+) for provider- and non-provider-specific questions. Chi-square (goodness-of-fit) analysis was performed between the number of patients who gave perfect satisfaction on all provider-specific questions and the number of patients who gave perfect satisfaction for non-provider scores irrespective of their provider-specific scoring. Finally, Spearman’s correlation coefficient matrixes were calculated to determine which specific elements of the PGOMPS survey correlated with a higher likelihood to recommend the practice.

## Results

We identified a total of 7138 unique new patient visits during our study period. Twenty-four were excluded for English as a second language. Five thousand one hundred fourteen patient visits seen by 41 different providers were included in our final analysis. Table 1 shows the breakdown of patient demographics and subspecialties. Our cohort was 90% White/Caucasian, had a total of 3008 (58%) females, and the average age was 48.02 ± 20.92. Additional baseline patient characteristics are illustrated in Table 1.

**Table 1:** Patient demographics

Age	
< 18	677
18–29	432
30–39	586
40–49	618
50–59	1032
60–69	1189
70+	629
Race	
Not Hispanic White/Caucasian	4538 (88%)
Hispanic/Latino	177 (3%)
African American/Black	29 (0.5%)
American Indian/Alaska Native	25 (0.5%)
Asian	59 (1%)
Other/choose not to disclose	335 (6%)
Sex	
Female	3008 (58%)
Male	2155 (42%)
Subspecialties	
Adult reconstruction	496
Foot and ankle	791
Hand	985
Non-operative	816
Oncology	50
Pediatrics	257
Spine	453
Sports med	1000
Trauma	315

A total of 1420 (26.5%) patients achieved perfect satisfaction on the total score. The frequency of patients who reported perfect satisfaction to all provider-specific questions was 43.9%. The frequency of patients who reported perfect satisfaction on non-provider questions was 19.10%. Chi-square analysis demonstrated that the proportion of patients reporting satisfaction with their provider was significantly greater than that for the non-provider elements of their visit (*p* <  0.001).

Table 2 illustrates the frequency in which perfect satisfaction was reported for each of the 25 PGOMPS questions. Of the top-scoring 5 questions, 4 were related directly to the provider: MD friendliness/courtesy (80.36%), MD spoke using clear language (80.35%), MD likelihood to recommend (78.80%), and MD confidence (78.74%). The 5 question patients were least satisfied with related to the practice in general: information about delays (53.80%), wait time (54.63%), ability to get desired appointment (59.50%), ease of getting on the phone (59.72%), and convenience of office hours (60.44%). The frequency of perfect satisfaction for provider and non-provider-specific questions based on sex and age is provided in Table 3h. Both female and male patients at all age groups were more significantly and more likely to be satisfied with provider-specific questions than they were with non-provider-specific questions.

**Table 2:** Frequency of perfect satisfaction for individual PGOMPS questions

MD friendliness/courtesy^‡^	80.36%
MD spoke using clear language^‡^	80.35%
Likelihood recommend practice	79.11%
MD likelihood to recommend^‡^	78.80%
MD confidence^‡^	78.74%
Cleanliness of practice	78.58%
MD explained problem or condition^‡^	77.26%
Nurse friendliness	77.19%
Courtesy registration staff	76.64%
MD concern for your questions/worries^‡^	76.30%
Staff work together	75.47%
MD effort to include you in decisions^‡^	75.44%
Concern for privacy	73.97%
Sensitivity to needs	71.67%
Staff protect safety	71.27%
Nurse concern	71.23%
MD instructions follow-up care^‡^	71.01%
MD information about meds^‡^	70.98%
MD time spent^‡^	70.11%
Ease of scheduling appointment	65.03%
Convenience of office hours	60.44%
Ease of getting on phone	59.72%
Ability to get desired appointment	59.50%
Wait time	54.63%
Information about delays	53.80%

*PGOMPS* Press Ganey Outpatient Medical Practice Survey

^‡^Provider-specific questions

**Table 3:** Frequency analysis of provider and non-provider specific PGOMPS questions by sex and age

Sex	Age	Sample (*n*)	Provider specific	Non-provider specific	*p* value
Female	< 18	350	42	13.71	< 0.001
	18–29	237	35.02	13.92	< 0.001
	30–39	340	43.53	18.53	< 0.001
	40–49	372	43.82	20.7	< 0.001
	50–59	622	44.53	18.49	< 0.001
	60–69	718	44.85	18.94	< 0.001
	70+	369	44.99	19.78	< 0.001
	All females	3008	43.42	18.12	< 0.001
Male	< 18	327	45.87	17.43	< 0.001
	18–29	195	40	18.97	< 0.001
	30–39	246	44.72	22.76	< 0.001
	40–49	246	48.37	23.98	< 0.001
	50–59	410	46.58	24.15	< 0.001
	60–69	471	42.89	17.62	< 0.001
	70+	260	42.31	19.23	< 0.001
	All males	2155	44.54	20.46	< 0.001

*PGOMPS* Press Ganey Outpatient Medical Practice Survey

Overall, provider-specific questions demonstrated a higher correlation with a patient’s likelihood of recommending the practice to others than questions related to the office and nursing staff, access of care, and moving through the visit. Out of the 10 questions that most correlated with recommending the practice, 8 of them were provider-specific (Table 4).

**Table 4:** Correlation of individual questions to “likelihood of your recommending our practice with others”

Question	Spearman’s coefficient	*p* value
Staff work together	0.79	< 0.001
MD likelihood to recommend	0.79	< 0.001
MD confidence	0.74	< 0.001
Sensitivity to needs	0.71	< 0.001
MD effort to include you in decisions	0.69	< 0.001
MD concern for your questions/worries	0.68	< 0.001
MD explained problem or condition	0.67	< 0.001
MD friendliness/courtesy	0.66	< 0.001
MD spoke using clear language	0.65	< 0.001
MD time spent	0.65	< 0.001
MD instructions follow-up care	0.65	< 0.001
Concern for privacy	0.64	< 0.001
MD information about meds	0.64	< 0.001
Cleanliness of practice	0.63	< 0.001
Nurse friendliness	0.57	< 0.001
Nurse concern	0.57	< 0.001
Staff protect safety	0.57	< 0.001
Courtesy registration staff	0.48	< 0.001
Ease of scheduling appointment	0.46	< 0.001
Convenience of office hours	0.45	< 0.001
Information about delays	0.45	< 0.001
Wait time	0.45	< 0.001
Ease of getting on phone	0.41	< 0.001
Ability to get desired appointment	0.41	< 0.001

## Discussion

Our primary finding is that patients experienced greater satisfaction with provider-specific Press Ganey questions than for non-provider questions pertaining to other elements of their clinic visit including ease of scheduling, nursing staff issues, and courtesy of the front desk staff. Our secondary finding also demonstrated that provider-specific questions had a stronger correlation with the likelihood of a patient to recommend the practice compared with the other elements of the survey. This finding is consistent with the findings of Chen et al., who evaluated the correlation between individual PGOMPS questions and the “likelihood of your recommending our practice to other” and “likelihood of your recommending this provider to others.” For both questions, provider-specific questions had a statistically significant positive correlation, whereas the other question categories had statistically significant negative correlations [28]. Similar findings have also been observed in the pediatric population [29–31].

Not surprisingly, our findings are consistent with previous literature that has demonstrated wait time to have significant influence on patient satisfaction scores [26, 32, 33]. There is discordance in the literature as to exactly how a patient’s wait time influences satisfaction scores. Patterson et al. demonstrated that time spent with provider was more impactful on patient satisfaction scores than actual wait time [34]. Rane et al., however, found that the likelihood of achieving patient satisfaction decreased by 3% for each additional minute of wait time [26]. Interestingly, we found “information about delays” to be less likely to receive perfect satisfaction than “wait time.” Although the aim of this study was not to determine how controlling for “information about delays” would influence the correlation between wait time and patient satisfaction scores, utilizing this knowledge could potentially help mitigate the seemingly inevitable negative impact that wait time has on patient satisfaction scores.

Patient satisfaction surveys can provide insight into factors that patients deem important in their care. Our study provides data showing the areas where orthopedic surgeons are receiving least perfect satisfaction scores. This allows these providers and orthopedic health care administrators to focus on specific areas of improvement. The three provider-specific questions least likely to receive perfect satisfaction were “time spent with MD,” “MD information about medications,” and “MD instructions on follow-up care.” Orthopedic surgeons seeking to improve their patient’s overall experience could potentially focus on these areas to improve patient satisfaction scores. Surgeons could lobby for longer appointment times for new patient visits and ensure they spend adequate time addressing the needs and questions of their patients. They could also focus efforts on giving clear instructions and information about medications and follow-up visits. Additionally, knowing that “information about delays” and “wait time” were the two questions least likely to receive prefect satisfaction, a health care system may consider hiring additional staff or provide training to current office staff with the goal of improving communication about the length of time a patient can expect before seeing their provider.

Our findings and previous literature demonstrate that overall, patients are satisfied with the orthopedic providers [28–31]. Areas where patients are demonstrating less satisfaction are with regard to the access of care and moving through their visit. These factors may not be directly under the control of the provider and should also be taken into account with regard to if and how patient satisfaction scores are attributed to physician reimbursement rates.

Our study has several limitations worth mentioning. Caution should be used with generalizing our study outside of orthopedics. Furthermore, our cohort was 90% White/Caucasian and would likely not be representative of other patient populations. Our study was performed at a single institution, which may limit the generalization of our results to other health care systems with differing demographic patient population makeup. Additionally, our institution treats patients from a large geographical distribution. Due to this, some patients may commute several hours to receive care. This may impact patient expectations, and therefore satisfaction, with the clinic encounter in a way that is not applicable to health care systems with smaller catchment areas. Another limitation of our study was non-response bias, which is likely naturally inherent to PGOMPS. Previous work has shown response rates ranging from 8.9 to 16.5% [26, 27]. Previously, a study found that the age, sex, insurance type, and orthopedic subspecialty of patients who completed the PGOMPS differed from non-responder patients [35]. It remains unclear how these factors may influence our results. Nonetheless, a low response rate limits the real-world application of the survey, and yet, it is often utilized by some health care systems to serve as a surrogate for “quality” or alter provider payments without regard to these limitations.

Although in our study we demonstrate that there is a statistically significant difference between provider-specific and non-provider-specific questions, we did not determine the impact of each question on overall PGOMPS scores. The aim of our study was to provide descriptive analysis of which questions orthopedic providers are likely to receive perfect satisfaction. As such, we did not control for any known factors that have been shown to affect patient satisfaction.

Another limitation to our study is that we only included first visit encounters. Evaluation of postoperative and/or follow-up visits may show different results. Tisano et al. demonstrated that established patients were more likely to be satisfied than new patients [36]. An analysis of how the responses to individual PGOMPS questions alter throughout the course of orthopedic follow-up would be informative.

## Conclusion

Our results suggest that the majority of patients who complete the Press Ganey Outpatient Medical Practice Survey are satisfied with their provider, demonstrating that room for improvement is limited with provider-specific portions of the clinic interaction. The majority of dissatisfaction pertains to aspects of the clinic that may not be directly within the control of providers. Administrators and leaders of health care teams should consider these results when seeking ways to improve patient satisfaction scores and when determining how and if scores should be linked to reimbursements.

## Data Availability

We do not feel that it is appropriate to publish the raw data used to perform this study, as it is largely proprietary and administered by a private corporation. The data can be available from the authors upon request.

## Abbreviation

PGOMPS: Press Ganey® Outpatient Medical Practice Survey
